# Supplementary material for: Limited impact of fingolimod treatment during the initial weeks of ART in SIV-infected rhesus macaques
Source: Nat Commun. 2022 Aug 27;13:5055. doi: 10.1038/s41467-022-32698-y (PMC9420154; doi:10.1038/s41467-022-32698-y)
Supplement: Supplementary file 1 — Supplementary information [file 41467_2022_32698_MOESM1_ESM.docx]

**Supplementary Information**

**Supplementary Figure 1. Blood T-cell populations during FTY720 treatment are relatively enriched in memory T-cells with an activated and cycling phenotype.**

**a.** Frequencies of **a-c.** CD4+ T cells and **d-f.** CD8+ T-cells with a **a,d.** memory (CD95+), **b,e.** activated (HLA-DR+), and **c,f.** cycling (Ki67+) phenotype in PBMCs from early FTY720 (red, n=8 RMs) or control (black, n=14 RMs) SIV-infected RMs. ART, antiretroviral treatment, is indicated with grey background. FTY720 treatment is indicated with red background. **g.** Representative flow cytometry staining for the different subsets of interest. Data are presented as the mean ± SD. Statistical differences between FTY720 treated and control groups are indicated in asterisks and were assessed with a two-sided (95% CI) Mann-Whitney u-test.

**Supplementary Figure 2. Absolute counts of cytolytic CD8+ T-cells, NK, monocytes, and B-cells are reduced during FTY720 treatment.**

**a,b.** Frequencies and **c,d.** absolute counts of CD8+ T-cells expressing **a,c**. granzyme B (GrB+) and **b,d.** perforin (Perf+) at different weeks on treatment. Absolute counts of **e.** NK (NKG2A+CD8+), **f.** monocytes, and **g.** B cells in blood of early FTY720 (red, n=8 RMs) or control (black, n=14 RMs) SIV-infected RMs at different weeks on treatment. ART, antiretroviral treatment, is indicated with grey background. FTY720 treatment is indicated with red background. Data are presented as the mean ± SD. Statistical differences between FTY720 treated and control groups are indicated in asterisks and were assessed with a two-sided (95% CI) Mann-Whitney u-test.

**Supplementary Figure 3. Blood T-cell population levels remain invariable at the latest on-ART experimental points, up to 10 months after the last dose of FTY720.**

**a-c.** Frequencies and **d-f.** absolute counts of blood CD3+, CD4+, and CD8+ T-cells at several weeks post infection during antiretroviral treatment (ART). Frequencies of **g-i.** CD4+ and **j-l.** CD8+ T-cells with a **g,j.** memory (CD95+), **h,k.** activated (HLA-DR+), and **i,l.** proliferating (Ki67+) phenotype in peripheral blood mononuclear cells (PBMCs) from early FTY720 (red, n=8 RMs) or control (black, n=6 RMs) SIV-infected RMs during ART. ART, antiretroviral treatment, is indicated with grey background. Data are presented as the mean ± SD. Statistical differences between FTY720 treated and control groups were assessed with a two-sided (95% CI) Mann-Whitney u-test.

**Supplementary Figure 4. Frequencies of B cells, monocytes, and NK cells in lymph nodes are similar between treatment groups throughout FTY720 treatment.**

Frequencies of **a.** B cells, **b.** monocytes, and **c.** NK cells (NKG2A+CD8+) in lymph node (LN) from early FTY720 (red, n=8 RMs) or control (black, n=14 RMs) SIV-infected RMs at -1 and 8 weeks on treatment. ART, antiretroviral treatment, is indicated with grey background. FTY720 treatment is indicated with red background. Data are presented as the mean ± SD. Statistical differences between FTY720 treated and control groups were assessed with a two-sided (95% CI) Mann-Whitney u-test.

**Supplementary Figure 5. Flow cytometry gating strategy used to sort Tfh and non-Tfh memory CD4+ T-cells from lymph node (LN).**

**Supplementary Figure 6. Virologic parameters were comparable between the 6 controls that underwent ATI and the other 8 animals included in the control group.**

Viral loads (copies/mL) at **a.** day 14 and **b.** day 42 post-infection (pre-ART) as well as **c.** lengths (in months) of viral load suppression during ART are compared between the control animals that underwent ATI (n=6 RMs; black), the control animals that did not undergo ATI (n=8 RMs; green), and the early FTY720 treated animals that underwent ATI (n=8 RMs; red). Data are presented as the mean ± SEM. Statistical differences between FTY720 treated and control groups were assessed with a two-sided (95% CI) Mann-Whitney u-test.

**Supplementary Tables**

**Table 1. Characteristics of the animals included in the study.**

**Table 2. Primer and probe sequences used in the ddPCR assay to quantify intact SIV genomes.**

| **PCR** | **Prime/Probe Name** | **Position** | **Sequence** | **Fluorophore** | **Quencher** |
| --- | --- | --- | --- | --- | --- |
| SIV_239_ IPDA pol | polF | 4901-4922 | GCAGGGATAGAGCACACCTTTG | N/A | N/A |
|  | polR | 5003-5030 | CTATGGTTTCTACTGAATTTGCTTGTTC | N/A | N/A |
|  | pol intact probe | 4965-4981 | TTTCAGGTGGTGATTCA | FAM | MGBNFQ |
|  | pol hyper probe | 4963-4978 | TAGGTGGTGATTTATT | None | MGBNFQ |
| SIV_239_ IPDA env | envF | 6866-6893 | CCTCAATAAAGCCTTGTGTAAAATTATC | N/A | N/A |
|  | envR | 6942-6965 | GTTGTTATTGATTTTGTCAATCCC | N/A | N/A |
|  | env intact probe | 6901-6919 | TGCATTACTATGAGATGC | VIC | MGBNFQ |
|  | env hyper probe | 6901-6919 | TGCATTACTATAAAATGC | None | MGBNFQ |
| RM RPP30 | 1F RPP30 |  | AGGATGCTCCGGGAGTATGTA | N/A | N/A |
|  | 1R RPP30 |  | CCTGCTTGTCACCTATATAACAT | N/A | N/A |
|  | 1 RPP30 probe |  | TCAAGCTGGGAGACGGAAGAGTCAGT | FAM | ZEN/IABkFQ |
| RM RPP30 | 2F RPP30 |  | ACAGACTCACACAATTTAGG | N/A | N/A |
|  | 2R RPP30 |  | ACATTCATGCCACTGCACTC | N/A | N/A |
|  | 2 RPP30 probe |  | ACAGGGTCTCACTTTGTTGTCCA | HEX | ZEN/IABkFQ |
| SIV_239_ 2-LTR | F 2-LTR |  | CGCCTGGTCAACTCGGTACTC | N/A | N/A |
|  | R 2-LTR |  | GGTATGATGCCTTCTTCCTTTTCTAAG | N/A | N/A |
|  | 2-LTR probe |  | CCCTGGTCTGTTAGGACCCTTTCTGCTTTG | FAM | MGBNFQ |

**Table 3. PCR conditions used in the ddPCR assay to quantify intact SIV genomes.**

| **Temperature** | **Time** | **Cycles** |
| --- | --- | --- |
| 95º C | 10 min | x 1 |
| 94º C | 30 sec | x 40 |
| 53º C | 1 min |  |
| 98º C | 10 min | x 1 |
| 12º C | ∞ |  |
