# Supplementary material for: Limited impact of fingolimod treatment during the initial weeks of ART in SIV-infected rhesus macaques
Source: Nat Commun. 2022 Aug 27;13:5055. doi: 10.1038/s41467-022-32698-y (PMC9420154; doi:10.1038/s41467-022-32698-y)
Supplement: Supplementary file 3 — Reporting Summary [file 41467_2022_32698_MOESM3_ESM.pdf]

# nature portfolio

Corresponding author(s): *Double-blind peer review submissions: write DBPR and your manuscript number here instead of author names.*

Last updated by author(s): *YYYY-MM-DD*

# Reporting Summary

Nature Portfolio wishes to improve the reproducibility of the work that we publish. This form provides structure for consistency and transparency in reporting. For further information on Nature Portfolio policies, see our Editorial Policies and the Editorial Policy Checklist.

## Statistics

For all statistical analyses, confirm that the following items are present in the figure legend, table legend, main text, or Methods section.

| n/a | Confirmed | |
|---|---|---|
| ☐ | ☒ | The exact sample size (*n*) for each experimental group/condition, given as a discrete number and unit of measurement |
| ☐ | ☒ | A statement on whether measurements were taken from distinct samples or whether the same sample was measured repeatedly |
| ☐ | ☒ | The statistical test(s) used AND whether they are one- or two-sided<br>*Only common tests should be described solely by name; describe more complex techniques in the Methods section.* |
| ☐ | ☒ | A description of all covariates tested |
| ☐ | ☒ | A description of any assumptions or corrections, such as tests of normality and adjustment for multiple comparisons |
| ☐ | ☒ | A full description of the statistical parameters including central tendency (e.g. means) or other basic estimates (e.g. regression coefficient) AND variation (e.g. standard deviation) or associated estimates of uncertainty (e.g. confidence intervals) |
| ☐ | ☒ | For null hypothesis testing, the test statistic (e.g. *F*, *t*, *r*) with confidence intervals, effect sizes, degrees of freedom and *P* value noted<br>*Give P values as exact values whenever suitable.* |
| ☒ | ☐ | For Bayesian analysis, information on the choice of priors and Markov chain Monte Carlo settings |
| ☒ | ☐ | For hierarchical and complex designs, identification of the appropriate level for tests and full reporting of outcomes |
| ☒ | ☐ | Estimates of effect sizes (e.g. Cohen's *d*, Pearson's *r*), indicating how they were calculated |

*Our web collection on statistics for biologists contains articles on many of the points above.*

## Software and code

Policy information about availability of computer code

| | |
|---|---|
| Data collection | Flow cytometric acquisition was performed on a BD LSRII Flow Cytometer driven by BD FACSDiva software version 9.0. Analysis of the acquired data was performed by FlowJo software version 10.8.1. (Tree Star Inc.). |
| Data analysis | Data analyses were performed using GraphPad Prism version 9.4.1. (GraphPad Software, Inc., La Jolla, CA). |

For manuscripts utilizing custom algorithms or software that are central to the research but not yet described in published literature, software must be made available to editors and reviewers. We strongly encourage code deposition in a community repository (e.g. GitHub). See the Nature Portfolio guidelines for submitting code & software for further information.

## Data

Policy information about availability of data

All manuscripts must include a data availability statement. This statement should provide the following information, where applicable:

- Accession codes, unique identifiers, or web links for publicly available datasets
- A description of any restrictions on data availability
- For clinical datasets or third party data, please ensure that the statement adheres to our policy

The raw data for all graphs generated in this study are provided in the Supplementary Information/Source Data file.

## Human research participants

Policy information about studies involving human research participants and Sex and Gender in Research.

| | |
|---|---|
| Reporting on sex and gender | Not applicable. |
| Population characteristics | Not applicable. |
| Recruitment | Not applicable. |
| Ethics oversight | Not applicable. |

Note that full information on the approval of the study protocol must also be provided in the manuscript.

# Field-specific reporting

Please select the one below that is the best fit for your research. If you are not sure, read the appropriate sections before making your selection.

☒ Life sciences ☐ Behavioural & social sciences ☐ Ecological, evolutionary & environmental sciences

For a reference copy of the document with all sections, see nature.com/documents/nr-reporting-summary-flat.pdf

# Life sciences study design

All studies must disclose on these points even when the disclosure is negative.

| | |
|---|---|
| Sample size | n=22 individual rhesus macaques total; Group sizes (n=8 for early FTY720, n=14 for controls) were selected to provide approximately 80% power to detect mean differences of 1.5 standard deviations or greater.<br>Results are expressed as the mean ± SD. Statistical significance of immunophenotyping and viral data between time points and study groups were performed using a paired or two-sided (95% CI) Mann-Whitney unpaired u-test when appropriate. A P value less than 0.05 was considered statistically significant, and indicated with exact p-values in the graphs. |
| Data exclusions | For cell-associated SIV-DNA and -RNA measurements, data were excluded for samples if <10,000 cells were sorted and values fell outside the assay limit of detection. This threshold for exclusion was predetermined based on the assays limit of detection in conjunction with the number of replicates.<br>Select flow cytometry data points were excluded based on indications of aberrant staining. |
| Replication | No attempts were made to replicate data, as this is a long term in vivo study with substantial costs pertaining to animal acquisition and care. Sorted LN cells were subsequently purity sort (>96%) to confirm phenotypic identity. |
| Randomization | At ART introduction (6 wpi), the 22 animals were assigned to two treatment groups comparable in term of age, weight, peak, and set-point viremia: untreated animals (control group, 14 RMs) and FTY720 treated animals (FTY720, 8 RMs). All animals in the cohort were female (Supplementary Table 1). Animals were negative for Mamu-B*08, and Mamu-B*17, known protective alleles in the RM model of SIV infection. Animals continued on-ART for 12 months, and viral rebound was followed for additional 4 months after analytic treatment interruption (ATI). During this phase of the study, 8 animals from the untreated group were redirected towards a different study, for which they underwent additional immune interventions; thus, for the ATI phase the study includes 6 control animals and 8 FTY720 treated animals. Of note, all the key immunologic and virologic parameters measured in the study were comparable between the 6 animals that underwent ATI and the other 8 controls that were redirected to a different study design (Supplementary Figure 6). At the end of the ATI follow-up, all animals underwent necropsy. |
| Blinding | Blinding of primary investigators was not possible as we were responsible of the distribution of compounds for administration and stratification of the animals into treatment groups. Secondary collaborators were blinded to treatment groups prior to analysis of shipped materials. |

# Reporting for specific materials, systems and methods

We require information from authors about some types of materials, experimental systems and methods used in many studies. Here, indicate whether each material, system or method listed is relevant to your study. If you are not sure if a list item applies to your research, read the appropriate section before selecting a response.

| Materials & experimental systems | |
|---|---|
| n/a | Involved in the study |
| ☐ | ☒ Antibodies |
| ☐ | ☒ Eukaryotic cell lines |
| ☒ | ☐ Palaeontology and archaeology |
| ☐ | ☒ Animals and other organisms |
| ☒ | ☐ Clinical data |
| ☒ | ☐ Dual use research of concern |

| Methods | |
|---|---|
| n/a | Involved in the study |
| ☒ | ☐ ChIP-seq |
| ☐ | ☒ Flow cytometry |
| ☒ | ☐ MRI-based neuroimaging |

## Antibodies

Antibodies used

18-parameter flow cytometric analysis was performed on fresh PBMCs, and mononuclear cells (1x106 cells) derived from LN according to standard procedures using a panel of monoclonal antibodies that we and others, have shown to be cross-reactive in RMs50,51. Abs were used as per manufacturer's recommendations: anti-CD95-PE-Cy5 (clone DX2, 10 µL, cat #559773), anti-Ki-67-Alexa Fluor 700 (clone B56, 5 µL, cat #561277), anti-CD56-BV605 (clone B159, 5 µL, cat. #740405), anti-CD16-BV650 (clone 3G8, 5 µL, cat. #563692), anti-CD3-BUV395 (clone SP34-2, 5 µL, cat #564117), anti-CD8-BUV496 (clone RPA-T8, 5 µL, cat #564804), and anti-CD14-BUV737 (clone M5E2, 5 µL, cat #564444), all from BD Biosciences; anti-CD4-APC-Cy7 (clone OKT4, 5 µL, cat #317418), anti-CD20-PerCP-Cy5.5 (clone 2H7, 5 µL, cat #302326), and anti-HLA-DR-BV711 (clone L243, 5 µL, cat #307644), all from Biolegend; anti-NKG2a-APC (clone Z199, 5 µL, cat #A60797) from Beckman Coulter; anti-GrB-PE-Texas Red (clone GB11, 5 µL, cat #GRB17) and Aqua Live/Dead Fixable Aqua from Invitrogen (AmCyan, 2 µL of 1:20 PBS dilution, cat. L34957); anti-Perforin-FITC (clone Pf-344, 5 µL cat #3465-7) from MABTECH. Flow cytometric acquisition was performed on at least 100,000 CD3+ T-cells on a BD LSRII Flow Cytometer driven by BD FACSDiva software. Analysis of the acquired data was performed by FlowJo software (Tree Star Inc.).
FACS cell sorting
Mononuclear cells isolated from LN were stained with anti-CD3-APC-Cy7 (clone SP34-2, 5 µL, cat #557757), anti-CD28-PE-CF594 (clone CD28.2, 5 µL, cat #562296), and anti-CD95-PECy5 (clone DX2, 10 µL, cat #559773) from BD Biosciences; anti-CD4-BV650 (clone OKT4, 2 µL, cat #317436), anti-PD-1-BV421 (clone EH12.2H7, 5 µL, cat # 3299220), and anti-CD200-PE (clone OX-104, 5 µL, cat #329206) from Biolegend; anti-CD8-FITC (clone 3B5, 5 µL, cat #MHCD0801-4) from Thermo Fisher Scientific; and Aqua Live/Dead Fixable Aqua from Invitrogen (AmCyan, 2 µL of 1:20 PBS dilution, cat. L34957). Sorting of CD4+ Tfh (PD1+CD200hi) was performed using a FACS AriaII (BD Biosciences) in samples collected before, during FTY720 treatment, at ART interruption, and 29 days after ART interruption. Post-sorting FACS analysis determined that sorted CD4+ T cell subsets were on average >96% pure.

Validation

All antibodies (anti-human) are validated and annotated on their catalog page as human reactive by the manufacturers on their associated catalog page (they typically do not test/report for RM reactivity or are listed as potentially macaque reactive).
All mAbs used in this study were either previously verified as rhesus reactive in our previous studies (see references below) or were independently verified as rhesus-reactive via databases maintained by the NHP Reagent Resource. All mAbs have previously been internally validated via FMO tests.
McGary, C.S., Deleage, C., Harper, J., Micci, L., Ribeiro, S.P., et al. CTLA-4(+)PD-1(-) Memory CD4(+) T Cells Critically Contribute to Viral Persistence in Antiretroviral Therapy-Suppressed, SIV-Infected Rhesus Macaques. Immunity 47, 776-788 e775 (2017).
Micci, L., Ryan, E.S., Fromentin, R., Bosinger, S.E., Harper, J.L., He, T., Paganini, S., Easley, K.A., Chahroudi, A., Benne, C., et al. (2015). Interleukin-21 combined with ART reduces inflammation and viral reservoir in SIV-infected macaques. J Clin Invest 125, 4497-4513.

## Eukaryotic cell lines

Policy information about cell lines and Sex and Gender in Research

| | |
|---|---|
| Cell line source(s) | P815 mastocytoma ATCC TIB-64™ |
| Authentication | The cell line was not authenticated. |
| Mycoplasma contamination | All cell lines tested negative for mycoplasma contamination. |
| Commonly misidentified lines (See ICLAC register) | n.a. |

## Animals and other research organisms

Policy information about studies involving animals; ARRIVE guidelines recommended for reporting animal research, and Sex and Gender in Research

| | |
|---|---|
| Laboratory animals | 22 specific-pathogen free (SPF) Indian rhesus macaques (RMs; Macaca mulatta), were housed at the Yerkes National Primate Research Center (YNPRC), Atlanta, GA, as recently described. Animals were negative for Mamu-B*08, and Mamu-B*17, known protective alleles in the RM model of SIV infection. All animals were female and with ages at time of infection between 46 and 110 months. |
| Wild animals | Study did not involve wild animals. |

| | |
|---|---|
| Reporting on sex | All animals in the cohort were female. |
| Field-collected samples | Study did not involve field-collected samples. |
| Ethics oversight | All animal experimentations were conducted following guidelines established by the Animal Welfare Act and by the NIH's Guide for the Care and Use of Laboratory Animals, 8th edition. All procedures were performed in accordance with institutional regulations after review and approval by Emory University's Institutional Animal Care and Usage Committee (IACUC; Permit number YER2002876) at Yerkes National Primate Research Center (YNPRC). Animal care facilities are accredited by the U.S. Department of Agriculture (USDA) and the Association for Assessment and Accreditation of Laboratory Animal Care (AAALAC) International. |

Note that full information on the approval of the study protocol must also be provided in the manuscript.

# Flow Cytometry

## Plots

Confirm that:

☒ The axis labels state the marker and fluorochrome used (e.g. CD4-FITC).

☒ The axis scales are clearly visible. Include numbers along axes only for bottom left plot of group (a 'group' is an analysis of identical markers).

☒ All plots are contour plots with outliers or pseudocolor plots.

☒ A numerical value for number of cells or percentage (with statistics) is provided.

## Methodology

| | |
|---|---|
| Sample preparation | Flow cytometric analysis: Collections of blood, and LN, were performed longitudinally during the entire study and at the necropsy. Blood samples were used for a complete blood count (CBC). Plasma was separated from EDTA-anticoagulated blood by centrifugation within 1 hour of phlebotomy. From EDTA-anticoagulated blood, PBMCs were isolated using a Ficoll-Paque Premium density centrifugation (GE Healthcare), and washed with R10 media. R10 media was composed of RPMI 1640 (Corning) supplemented with 10% heat-inactivated fetal bovine serum (FBS, Corning), 100 IU/ml penicillin, 100 mg/mL streptomycin, and 200 mM L-glutamine (GeminiBio). For LN biopsies, the skin over the axillary or inguinal region was clipped and then surgically prepared. An incision was made in the skin over the LN, which was then exposed by blunt dissection and excised over clamps. Half of each LN biopsy was paraffin fixed for immunohistochemistry (IHC) analysis, while the other half was homogenized and passed through a 70-µm cell strainer to isolate lymphocytes and washed with R10 media. Mononuclear cells were counted for viability using a Countess II Automated Cell Counter (Thermo Fisher) with trypan blue stain. All samples were processed, stained, fixed (1% paraformaldehyde) and analyzed by flow cytometry within 24 hours of collection.<br>FACS cell sorting: Mononuclear cells isolated from LN were stained, and sorted. Post-sorting FACS analysis determined that sorted CD4+ T cell subsets were on average >96% pure. |
| Instrument | Flow cytometric acquisition was performed on a BD LSRII Flow Cytometer.<br>FACS cell sorting was performed using a FACS AriaII (BD Biosciences) |
| Software | Flow cytometric acquisition was performed with BD FACSDiva software. Analysis of the acquired data was performed by FlowJo software (Tree Star Inc.). |
| Cell population abundance | Flow cytometric acquisition was performed on at least 100,000 CD3+ T-cells.<br>FACS cell sorting was performed on the maximum of cells available in the sample and were purify sorted (>96% purity). |
| Gating strategy | Mononuclear cells were defined by laying on the diagonal of FSC-H versus FSC-A, and lymphocytes were gated from SSC-A versus<br>FSC-A. CD4+ and CD8+ T-cells were pre-gated as live CD3+, and memory (CD95+), activation (HLA-DR+), cycling (Ki67+), cytolytic (GrB, Perf+) markers were gated inside total CD4 and CD8 T-cell populations. NK cells were defined as CD3-, HLA-DR-, CD20-, NKG2A+, CD8+. NK cell subsets were identified as CD56 and CD16. Monocytes were defined as CD3-, HLA-DR+, CD20-, CD14 and CD16.<br>For phenotypic discrimination of sorted populations, TFH were defined as PD-1hiCD200hi.<br>Gating strategies are supplied as Supplementary Fig. 1 and 5. |

☒ Tick this box to confirm that a figure exemplifying the gating strategy is provided in the Supplementary Information.
